# From Rare Genetic Variants to Polygenic Risk: Understanding the Genetic Basis of Cardiomyopathies

**DOI:** 10.3390/jcdd12070274

**Published:** 2025-07-17

**Authors:** Ana Belen Garcia-Ruano, Elena Sola-Garcia, Maria Martin-Istillarty, Jose Angel Urbano-Moral

**Affiliations:** 1 Inherited Cardiac Conditions and Myocardial Diseases Unit, Cardiology Department, University Hospital Jaen, 23007 Jaen, Spain; 2 Genetics Section, Clinical Analysis Department, University Hospital Jaen, 23007 Jaen, Spain; 3 Cardiovascular Imaging Laboratory, Cardiology Department, University Hospital Jaen, 23007 Jaen, Spain

**Keywords:** cardiomyopathy, genetic architecture, genetic modifiers, incomplete penetrance, intermediate effect variants, monogenic inheritance, personalized medicine, polygenic risk

## Abstract

Cardiomyopathies represent a heterogeneous group of myocardial disorders, traditionally classified by phenotype into hypertrophic, dilated, and arrhythmogenic. Historically, these conditions have been attributed to high-penetrance rare variants in key structural genes, consistent with a classical Mendelian pattern of inheritance. However, emerging evidence suggests that this model does not fully capture the full spectrum and complexity of disease expression. Many patients do not harbor identifiable pathogenic variants, while others carrying well-known disease-causing variants remain unaffected. This highlights the role of incomplete penetrance, likely modulated by additional genetic modifiers. Recent advances in genomics have revealed a broader view of the genetic basis of cardiomyopathies, introducing new players such as common genetic variants identified as risk alleles, as well as intermediate-effect variants. This continuum of genetic risk, reflecting an overall genetic influence, interacts further with environmental and lifestyle factors, likely contributing together to the observed variability in clinical presentation. This model offers a more realistic framework for understanding genetic inheritance and helps provide a clearer picture of disease expression and penetrance. This review explores the evolving genetic architecture of cardiomyopathies, spanning from a monogenic foundation to intermediate-risk variants and complex polygenic contribution. Recognizing this continuum is essential for enhancing diagnostic accuracy, guiding family screening strategies, and enabling personalized patient management.

## 1. Introduction

Cardiomyopathies represent a heterogeneous group of myocardial disorders, traditionally classified by phenotype into hypertrophic (HCM), dilated (DCM), and arrhythmogenic (ACM). Historically, these conditions have been attributed to high-penetrance rare variants in key structural genes, consistent with a classical Mendelian pattern of inheritance. Most notably, variants in sarcomeric genes in HCM and desmosomal genes in ACM have generally been considered sufficient to cause disease, often with an autosomal dominant trait [1,2,3,4,5]. This classical model has driven significant advances in this field, including the identification of core cardiomyopathy genes such as *MYH7*, *MYBPC3*, *TTN*, *PKP2*, and *DSP*, the development of cascade family screening, and the integration of genetic findings into prognostic risk models [4,5,6]. However, emerging evidence suggests that this approach does not fully capture the entire spectrum and complexity of disease expression. A substantial proportion of patients—particularly those without a family history of cardiomyopathy or presenting with late-onset or milder phenotypes—do not harbor identifiable pathogenic variants in disease-related genes, whereas some individuals may carry well-established disease-causing variants yet remain clinically unaffected [3,7]. This apparent discrepancy is further exemplified by the marked intrafamilial phenotypic variability observed even among carriers of the same pathogenic variant, pointing to the influence of modifier genes, environmental factors, or epigenetic mechanisms [4,6]. In addition, a subgroup of patients with severe disease and clear familial clustering remains without a genetic diagnosis, suggesting that non-Mendelian factors or yet unidentified genetic contributors may also play a role [7]. Thus, recent advances in genomics have revealed a broader understanding of the genetic basis in regard to cardiomyopathies, with common genetic variations emerging as risk alleles, along with intermediate-effect variants. Common genetic variations, particularly single-nucleotide polymorphisms (SNPs) identified through genome-wide association studies (GWAS), have been found to be able to significantly alter disease susceptibility and severity [8,9]. On the other hand, intermediate-effect variants—more frequent than Mendelian variations but less common than SNPs identified as polygenic risk alleles—confer modest risk and low penetrance yet may act additively or synergistically with other variants. These genetic changes are increasingly recognized within an oligogenic inheritance model, in which multiple variants collectively contribute to disease expression. This framework reflects a growing shift away from monogenic causality toward a complex genetic architecture underlying phenotypic expression in cardiomyopathies [1,10].

**Figure 1:**
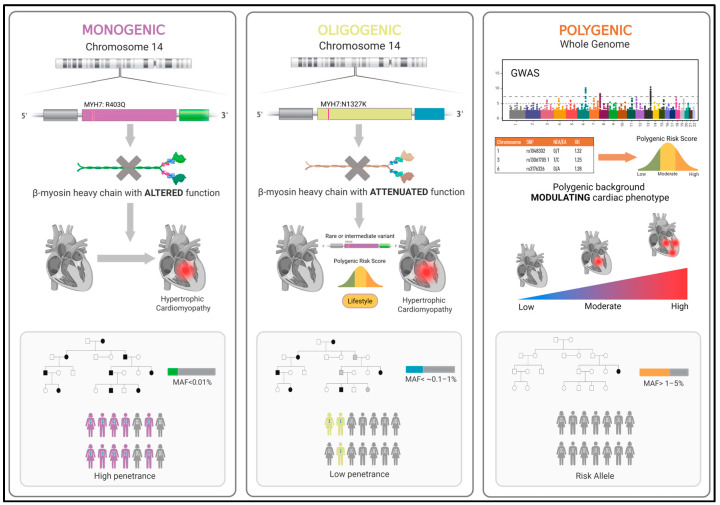
The genetic continuum of cardiomyopathies: From monogenic to polygenic inheritance. This figure illustrates the genetic spectrum of cardiomyopathies, from rare, high-impact to common polygenic variants. The monogenic model (**left**) involves rare pathogenic variants with high penetrance and autosomal dominant inheritance (e.g., *MYH7*:p.R403Q). These variants (minor allele frequency [MAF] < 0.01%) directly alter protein structure and are typically sufficient to cause disease. The oligogenic model (**center**) includes intermediate-effect variants (e.g., *MYH7*:p.N1327K), which interact with other genetic and environmental factors. These variants have moderate frequency (MAF ~0.1–1%), incomplete penetrance, and variable expressivity, often requiring additional modifiers for disease manifestation. The polygenic model (**right**) reflects the cumulative contribution of numerous common variants (MAF > 1%) with small individual effects. These variants act additively and probabilistically, are quantified using polygenic risk scores (PRS), and follow a complex, non-Mendelian inheritance pattern. While each variant confers minimal risk, their combined burden can stratify individuals by susceptibility. PRS may influence disease expressivity in monogenic cases and help explain phenotypes in remainder individuals. Although examples are drawn from HCM, this continuum applies broadly to other forms such as DCM and ACM, highlighting a shift toward an integrated understanding of genetic risk.

Accordingly, cardiomyopathies are increasingly viewed, from a genetic perspective, as existing along a continuum of genetic risk: from high-penetrance rare variants at one end to common variants associated with modest disease risk at the other, with intermediate-effect variants bridging the spectrum. This model, understood as an overall genetic influence on individuals, further interacts with environmental and lifestyle factors, which together likely underlie the observed variability in clinical presentation. It represents a more comprehensive and realistic model of genetic inheritance, enabling a clearer understanding of disease expression and penetrance. The present review explores the evolving genetic architecture of cardiomyopathies, spanning from a monogenic foundation to intermediate-risk variants and complex polygenic contributions (Figure 1). Recognizing this continuum is crucial for enhancing diagnostic accuracy, guiding family screening strategies, and enabling personalized patient management.

## 2. Rare High-Impact Variants: Monogenic Basis of Cardiomyopathies

High-penetrance rare variants have historically formed the cornerstone of genetic causality in inherited cardiomyopathies. These variants are typically protein-altering—i.e., missense, nonsense, frameshift, or splice-disrupting variations—, exhibit very low allele frequencies in the general population (typically <0.01%), and are often inherited in an autosomal dominant pattern, showing familial segregation [11]. In clinical genetics, a variant is generally classified as high-penetrance if more than 50% of carriers develop the associated phenotype during their lifetime, although this threshold can vary depending on the context and gene-specific behavior [12]. This threshold helps differentiate variants that are likely to cause disease independently from those that require additional genetic or environmental modifiers. In cardiomyopathies, high-penetrance variants typically act as strong monogenic drivers and have been the main focus of genetic testing and cascade screening over the past two decades (Table 1) [1,3,4].

In HCM, pathogenic variants in sarcomeric genes are the main cause of disease, with *MYBPC3* and *MYH7* being most commonly implicated [21]. *MYBPC3* variants are often truncating and typically lead to late-onset disease (over 40 years old) [21]. This large gene, which contains 35 exons and extensive intronic regions, encodes cardiac myosin-binding protein C, a key regulator of sarcomere function. Truncating variants result in haploinsufficiency, impairing sarcomeric performance and contributing to diastolic dysfunction [22]. Additionally, deep intronic *MYBPC3* variants can activate cryptic splice sites, leading to pseudoexon inclusion and premature stop codons, further supporting haploinsufficiency as a central pathogenic mechanism [23]. In contrast, *MYH7* variants are predominantly missense and are usually associated with earlier-onset disease [21]. *MYH7* encodes the β-myosin heavy chain, a crucial sarcomeric motor protein composed of a globular head (motor domain), neck, and coiled-coil tail. Variants often cluster in the head domain—recognized as a mutational hotspot—where they alter actin binding and ATPase activity, leading to hypercontractility [24]. Disease-causing variants in other sarcomeric genes are less frequent but well validated, with phenotypes ranging from mild hypertrophy combined with high arrhythmic risk (*TNNT2*) to apical or restrictive HCM (*TNNI3*) or early-onset concentric HCM as well as infantile or syndromic presentations (*MYL2* and *MYL3*) [25,26]. On the other hand, while several non-sarcomeric genes initially linked to HCM have been reclassified after expert re-evaluation, some remain clinically relevant in specific contexts. Biallelic truncating variants in *ALPK3* cause a syndromic, early-onset HCM/DCM phenotype with extracardiac features, while heterozygous variants may predispose to adult-onset HCM with reduced penetrance [27]. Other rare but potentially causative genes, including *CSRP3*, *FHOD3*, *FLNC*, *PLN*, *ACTN2*, *TRIM63*, and *KLHL24*, each account for less than 1–2% of cases and are often associated with atypical or overlapping phenotypes, such as combined hypertrophic and dilated features or skeletal muscle involvement in *FLNC*-related disease [28].

In DCM, truncating variants in *TTN* (TTNtv) represent the most prevalent genetic cause, identified in 11–15% of unselected DCM patients and up to 30–35% of familial cases, making *TTN* the primary contributor to the genetic architecture of the disease [3]. Other well-established DCM genes include *LMNA*, *RBM20*, *FLNC*, *DSP*, *BAG3*, *SCN5A*, and *PKP2*, each featuring distinct phenotypes [29,30]. Pathogenic variants in *LMNA* are associated with early-onset conduction disease, ventricular arrhythmias, and a high risk of sudden cardiac death [31]. Similarly, *RBM20*, a splicing regulator of titin, is typically linked to aggressive and early-onset DCM with arrhythmias, particularly in males [32]. Additionally, truncating variants in *FLNC* are implicated in myofibrillar disarray, skeletal muscle involvement, and recurrent ventricular arrhythmias, occasionally presenting with arrhythmic events that overlap with ACM [33]. Similar patterns have been observed with variants in *DSP*, *BAG3*, *SCN5A*, and *PKP2* [29,30]. Importantly, penetrance is incomplete and modulated by context: while TTNtv are typically pathogenic in affected individuals or symptomatic relatives, incidental findings in population studies show low penetrance and subtle structural abnormalities [34]. Some TTNtv are located in exons that are rarely included in the cardiac transcript—low proportion spliced-in (low-PSI)—or in meta-transcript–only regions, which correspond to parts of the gene present only in rare or minor isoforms not commonly expressed in the heart. Variants in these regions are often observed in healthy controls and may be tolerated or exert only mild effects, sometimes requiring a second pathogenic variant to cause disease. These low-PSI regions, such as parts of the I-band and certain Z-disk exons, undergo extensive alternative splicing and maintain the reading frame even if entire exons are skipped, reducing their pathogenic potential. In contrast, pathogenic TTNtv linked to DCM and other cardiomyopathies tend to cluster in constitutively expressed (high-PSI) exons, especially in the A-band and critical M-band regions important for sarcomere structure [35]. Environmental modifiers—including male sex, alcohol intake, peripartum stress, and exposure to cardiotoxic agents such as chemotherapy—further contribute to variable expressivity in genetically predisposed individuals [36]. Longitudinal data suggest a lifetime penetrance of only 13–17% in unselected TTNtv carriers, highlighting the need for context-aware interpretation and personalized management, and emphasizing that TTNtv act within a broader genetic–environmental interplay [29,34].

ACM is primarily associated with pathogenic variants in desmosomal genes such as *PKP2*, *DSP*, *DSG2*, *DSC2*, and *JUP*, which encode intercellular adhesion proteins whose disruption compromises desmosomal integrity and promotes fibrofatty infiltration and arrhythmogenesis [5]. *PKP2* and *DSP* encode plakophilin-2 and desmoplakin, essential for maintaining desmosomal function. Most pathogenic variants are loss-of-function (nonsense, frameshift, or splice-site), leading to reduced protein expression and impaired desmosomal structure and signaling. These defects cause myocardial cell detachment under mechanical stress, fibrofatty replacement, and increased arrhythmogenic risk, especially during adrenergic stimulation or exercise [37]. *PKP2* is the most frequently involved gene, identified in up to 40–50% of right-dominant forms of the disease, and is classically associated with exercise-induced arrhythmias [18,38]. Truncating variants in *DSP* are linked to biventricular or left-dominant forms and may present with cutaneous features [5]. Meanwhile, *FLNC* truncating variants have been found in overlapping DCM-ACM phenotypes characterized by frequent ventricular arrhythmias and skeletal muscle involvement [30]. Other genes include *LMNA*, linked to early-onset conduction disease and a high risk of malignant ventricular arrhythmias [31]; *SCN5A*, in arrhythmic phenotypes including Brugada syndrome and overlapping with DCM [29]; and *RBM20* truncating variants in aggressive DCM with high arrhythmic burden [32]. Similar to HCM and DCM, variant penetrance in ACM is incomplete, and phenotypic expression is modulated by both genetic and environmental factors. Notably, endurance exercise significantly increases the risk of phenotype expression and arrhythmic events in disease-causing variant carriers, particularly those with *PKP2* variants [39]. Emerging evidence also suggests a potential role of inflammation—either as a coexisting condition or triggered by infections or autoimmune processes—as an additional factor that may exacerbate disease expression and arrhythmic risk [40].

In summary, high-penetrance rare variants constitute the monogenic basis of cardiomyopathies. Each major subtype—HCM, DCM, and ACM—is associated with specific genes in which pathogenic variants can directly cause the disease. These variants typically confer substantial relative risks (odds ratios ranging from tens to hundreds) and often segregate within families. Their penetrance and phenotypic expression are often shaped by additional genetic and environmental factors. This insight has prompted increasing interest in the broader genetic architecture of cardiomyopathies—including intermediate-effect variants and polygenic risk—which will be explored in the following sections.

## 3. Intermediate Variants: Bridging the Gap in Genetic Risk

Within the genetic architecture of disease, intermediate-effect variants occupy a distinct position between high-penetrance, rare variants linked to monogenic inheritance and common variations with low individual effect sizes. Also known as moderate-penetrance alleles, these are more prevalent than classical Mendelian variants and confer a milder, yet still meaningful, increase in disease risk [41]. Individually, their effects are modest; however, when combined—particularly through interactions with other genetic or environmental factors—they can substantially influence disease susceptibility [41,42,43,44]. These intermediate variants represent a genetic middle ground, where risk is elevated but typically not sufficient to cause disease without additional modifiers.

This concept is well established in certain medical conditions. In lipid metabolism disorders and atherosclerosis, low-frequency variants in the *LPA* and *PCSK9* genes have moderate effects on individual risk but, because they are relatively common, they contribute substantially to the overall burden of disease at the population level [42]. Likewise, in hereditary cancer syndromes, moderate-penetrance variants in genes such as *CHEK2* or *ATM* are associated with a two- to fivefold increased risk of breast or ovarian cancer compared to the general population [43]. Moreover, many rare variants appear to fall into a gray area of moderate pathogenicity, prompting the use of quantitative tools—such as etiological fraction and variant clustering—to refine their clinical interpretation [44]. These observations are consistent with broader models of complex genetic risk.

The role of intermediate-effect variants in cardiomyopathies is gaining recognition. Recent large-scale studies have identified several sarcomeric variants that exhibit features consistent with modest penetrance (Table 2). Variants such as *TNNT2:p.R286H* and *TNNI3:p.R79C*, more prevalent in East Asian populations, have been associated with modestly increased risk of HCM, with estimated penetrance below 5% in the general population [45]. The *TNNT2:p.Asn271Ile* variant, linked to a low-risk, late-onset form of HCM, further illustrates the clinical relevance of sarcomeric variants with attenuated effects [46]. Additionally, a broader spectrum of rare variants in cardiomyopathy-associated genes, each with estimated penetrance below 10%, has been identified, supporting a model of inheritance that extends beyond strict monogenicity and informing the interpretation of secondary findings in genomic medicine [47].

A recent study identified a group of low-penetrance sarcomeric variants (LowSVs) associated with HCM [10]. Drawing on data from the SHaRe registry, 12 LowSVs were characterized by relatively high population frequencies (carrier frequency ~1:350), minor allele frequency above 5 × 10^−5^ (gnomAD database), and moderate enrichment among HCM patients (aggregate odds ratio ~15). These variants are generally linked to mild or late-onset forms of HCM when present alone, in contrast to high-penetrance variants. Indeed, many carriers may remain asymptomatic or develop only subtle features later in life, consistent with reduced penetrance. However, co-inheritance with classical pathogenic variants markedly worsens the clinical presentation: affected individuals present, on average, 11 years earlier and face more than double the risk of adverse events compared to those with only a pathogenic sarcomeric variant (hazard ratio ~2.0). Lifetime penetrance estimates suggest that LowSVs confer a risk of ~10% when identified through family screening, and only 1–2% when found incidentally, underscoring the strong context dependence of their pathogenicity.

These findings support an additive oligogenic model of genetic risk in cardiomyopathies, which may help explain the phenotypic variability observed among individuals carrying the same primary genetic alteration. In this model, additional LowSVs act as disease modifiers of disease expression. It also provides a framework to understand the lack of familial aggregation seen in mildly affected LowSV carriers, where other genetic and environmental factors likely play a role. It is important to emphasize that research on intermediate-effect variants in cardiomyopathies remains in its early stages, with HCM being the most thoroughly investigated to date. As the field advances, similar moderate-risk variants are expected to be identified in other cardiomyopathy subtypes. Variants in genes such as *LMNA* and *DSP* demonstrate variable expressivity: while truncating variants are well-established as pathogenic, certain missense variants have been linked to milder clinical phenotypes in DCM and ACM [3,29,30].

In summary, intermediate-effect variants highlight a fundamental and novel concept in cardiovascular genetics: pathogenicity exists along a continuum. Disease-associated variants do not simply have all-or-nothing outcomes; rather, their impact spans from highly deterministic to conditionally contributory, influenced by the surrounding genetic and environmental context. This perspective naturally leads into the realm of polygenic risk, where numerous common variants with individually small effects combine to influence overall disease susceptibility.

## 4. Polygenic Risk: Common Variations and Genetic Susceptibility

At the opposite end of the genetic spectrum from high-impact rare variants lies the domain of common genetic variations associated with increased disease risk. From these variations arises the concept of polygenic risk, which refers to the cumulative influence of numerous genetic variants—particularly SNPs identified in GWAS—each conferring a small individual effect on an individual’s susceptibility to disease. While any single common variant may confer only a marginal change in risk, their aggregate impact—typically quantified through the construction of polygenic risk scores (PRS)—can meaningfully stratify individuals across a spectrum of genetic susceptibility. In complex cardiovascular diseases such as coronary artery disease, assessing the associations between thousands to millions of SNPs and clinical phenotypes—as well as exploring their potential for clinical application—has become a major focus of interest in recent years [48]. The role of PRS in cardiomyopathies is dual: first, to estimate the risk of developing disease in monogenic variant-negative individuals; and second, to modulate disease expressivity in carriers of pathogenic variants [9,10]. Although these conditions often have a monogenic basis, growing evidence shows that common variants also influence disease risk and severity. In individuals without pathogenic variants, polygenic risk may represent the primary genetic driver [9], while in carriers, it can modulate penetrance and overall susceptibility [49].

HCM has traditionally been underrepresented in large-scale GWAS due to its relative rarity (~1 in 500 prevalence) and the strong influence of monogenic variants. However, this landscape has begun to shift through collaborative efforts aimed at assembling large, well-phenotyped cohorts. In 2021, one of the first robust GWAS in HCM analyzed over 2700 cases—including both sarcomeric variant carriers and non-carriers—against approximately 40,000 controls [8]. This study identified 12 genome-wide significant loci associated with HCM, several of which were located in or near genes with plausible roles in cardiac muscle function and disease pathogenesis (i.e., loci near *FHOD3* and *TTN*). The effect sizes of individual variants were modest, with a median per-allele odds ratio of ~1.25 (range: ~1.18 to 2.16 among genome-wide significant associations), consistent with findings in other complex diseases. Using these data, a PRS was developed, demonstrating that polygenic background, in combination with modifiable risk factors, contributes to both disease susceptibility and phenotypic expression. Notably, among individuals with HCM-causing variants, those with higher PRS exhibited more severe disease manifestations—such as increased left ventricular hypertrophy and earlier disease onset—whereas those with lower PRS had comparatively milder phenotypes [8]. These findings underscore the capacity of PRS to modulate the expressivity of monogenic HCM.

Expanding upon this area of research, the largest genetic study of HCM to date analyzed over 5900 cases from international cohorts, further advancing our understanding of the disease [9]. This study identified 34 genome-wide significant loci associated with HCM, including 15 novel signals, thereby substantially expanding the catalog of common variant associations (Table S1). Importantly, the analyses were stratified by sarcomeric variant status, distinguishing between sarcomere-positive and sarcomere-negative cases. Polygenic heritability—the proportion of phenotypic variance attributable to common variants—was found to be nearly twice as high in sarcomere-negative HCM compared to sarcomere-positive HCM [9]. These results support the long-standing hypothesis that sarcomere-negative HCM frequently arises from polygenic and multifactorial mechanisms, whereas pathogenic sarcomere-positive cases may require fewer additional risk alleles to cross the threshold for disease expression. These data also indicate that a high PRS may serve as a marker of increased HCM risk in individuals lacking a family history or identifiable pathogenic variants, thereby supporting the concept of a polygenic form of the disease.

DCM has also been shown to be influenced by polygenic factors. Using cardiac magnetic resonance imaging data from the UK Biobank, a PRS for DCM was developed in 2020 (Table S2) [34]. This score was associated with left ventricular dilation and reduced ejection fraction in the general population and was predictive of future DCM diagnoses. Notably, among carriers of TTNtv, those with higher PRS were significantly more likely to develop overt clinical DCM. Again, this finding highlights the role of polygenic burden as a modifier of penetrance in carriers of rare, high-impact variants, offering a potential explanation for the variable expressivity observed in these individuals.

Although PRSs hold promise for enhancing genetic risk stratification in cardiomyopathies, several challenges must be addressed before routine clinical application is feasible. Polygenic risk does not act in isolation: environmental and lifestyle factors—regular exercise or physical activity, hypertension, and other cardiovascular conditions—also modulate disease risk. One such factor is elevated diastolic blood pressure, which has been shown to significantly increase HCM risk, particularly among sarcomere-negative individuals [8]. Unlike rare pathogenic variants associated with monogenic forms of HCM, PRSs confer probabilistic risk and require careful calibration. Being in the top 5% of HCM PRS distribution, for instance, may increase absolute risk from approximately 0.2% to 1–2%, prompting important questions about clinical utility and appropriate follow-up strategies. Moreover, most PRSs have been derived from GWAS conducted in European-ancestry populations. Thus, predictive accuracy of these scores is limited in other groups due to genetic diversity and raises the need for multi-ancestry calibration [9,47]. Additional challenges arise in interpreting PRS in individuals already known to carry rare pathogenic variants. While a high PRS may indicate increased risk, current evidence is insufficient to warrant changes in clinical management based solely on polygenic background [8,10].

Looking ahead, integrated risk models that combine monogenic variants, polygenic scores, and non-genetic factors will be crucial for precise, individualized prediction of disease onset and progression. Despite current challenges, the path forward is evident: polygenic risk is moving from research into clinical practice in cardiomyopathies. Much like how polygenic scores complement lipid genes analysis to stratify risk in atherosclerotic, future cardiomyopathy clinics will likely integrate both rare variant status and polygenic burden to refine diagnosis, prognostication, and family counseling.

## 5. Overlap in Cardiomyopathies

Accurate interpretation of genetic findings in cardiomyopathies critically depends on a precise, multidisciplinary clinical diagnosis. However, defining clear phenotypic boundaries between different cardiomyopathies—particularly ACM and DCM—remains challenging. In some cases, ACM and DCM represent distinct disease entities; in others, they may lie on a shared pathological continuum [5,30]. This overlap is supported by genetic evidence: variants in *FLNC*, *LMNA*, and *DSP* have been implicated in both DCM and ACM phenotypes, often with variable degrees of arrhythmia and ventricular dysfunction [5,17,31,33]. For instance, truncating variants in *DSP* can manifest with left-dominant or biventricular ACM [5], while *FLNC* variants may present as a DCM with high arrhythmic burden [17]. Beyond gene overlap, variant type also plays a role in phenotypic expression. Missense and truncating variants in the same gene—such as *LMNA* or *TNNT2*—can lead to distinct clinical outcomes, ranging from conduction disease to aggressive arrhythmogenic forms [25,30]. These findings highlight the limitations of rigid phenotypic classifications and underscore the importance of integrative diagnostic approaches combining imaging, ECG features, family history, and genetics to interpret variants accurately.

## 6. Additional Risk Modulators

While cardiomyopathies have traditionally been considered Mendelian disorders caused by rare, high-penetrance variants, growing evidence reveals a more complex and heterogeneous genetic architecture. Many patients carry combinations of rare, low-effect, and common variants, suggesting oligogenic or polygenic contributions to disease onset and progression. In HCM, for example, the presence of multiple sarcomeric variants is associated with earlier onset and more severe disease, illustrating a genetic dosage effect [10]. In ACM, compound heterozygosity in desmosomal genes such as *PKP2* or *DSG2* can intensify disease severity [50]. Similarly, digenic inheritance, as seen in co-occurring TTNtv and *PLN* variants, has been linked to more severe forms of DCM [3].

Environmental and acquired factors further modulate phenotypic expression. High-intensity exercise has been shown to increase penetrance and arrhythmic risk in ACM, particularly in *PKP2* variant carriers [39], while external stressors such as chemotherapy or pregnancy may unmask DCM in individuals carrying TTNtv [36]. These gene–environment interactions contribute to the variable expressivity and incomplete penetrance frequently observed, even among carriers of the same variant. Moreover, common variants captured through PRS can influence cardiomyopathy risk and severity. PRS may act as the primary genetic driver in individuals without pathogenic monogenic variants or serve as modifiers of disease expressivity in variant carriers [9,10]. This complexity challenges the binary classification of “genotype-positive” and “genotype-negative” individuals, highlighting the need for a more refined and integrative approach to genetic risk assessment.

## 7. Future Directions

The evolving understanding of cardiomyopathy genetics is poised to transform clinical practice by integrating rare, intermediate-effect, and common genetic variants into personalized risk models. A major goal is the development of comprehensive risk calculators that combine rare pathogenic variants, moderate-risk variants, PRSs, and clinical data to provide individualized and precise risk assessments. PRSs for HCM and DCM are expected to complement current genetic and clinical markers in routine care. In parallel, ongoing research seeks to identify novel intermediate-risk variants and to quantify the contributions of gene-gene and gene-environment interactions, enabling more accurate estimates of disease penetrance and progression. Advances in functional genomics and multi-omics technologies are enhancing variant interpretation and supporting the validation of oligogenic mechanisms in experimental models. This integrated genetic approach represents a paradigm shift in cardiomyopathy management, moving from symptom-driven diagnosis toward proactive, risk-guided care. Comprehensive genetic profiling holds great potential to reduce the proportion of unexplained cases by facilitating earlier diagnosis and enhancing prognostic accuracy. Collectively, these developments are paving the way for personalized therapies tailored to each patient’s unique genetic architecture, ushering in a new era of targeted treatment.

## 8. Conclusions

Cardiomyopathies are increasingly recognized as genetically complex disorders influenced by the combined effects of rare, intermediate-effect, and common genetic variants. Rather than conforming strictly to a Mendelian inheritance model, most cases fall along a continuum of genetic susceptibility, reflecting a diverse spectrum of genetic complexity. This evolving paradigm challenges the classical notion of cardiomyopathies as purely monogenic disorders. It also carries important implications for diagnostic strategies, which are shifting from the identification of single pathogenic variants toward broader assessments of genetic architecture and cumulative risk. As a result, new terminology and personalized management strategies are emerging, aimed at capturing the multifactorial nature of disease expression by integrating monogenic, oligogenic, and polygenic contributions into an individualized model of care.

## Figures and Tables

**Table 1:** Estimated Penetrance in Selected Genes Associated with Cardiomyopathies. *MYBPC3*: myosin-binding protein C; HCM: hypertrophic cardiomyopathy; *MYH7*: beta-myosin heavy chain; TNNT2: Cardiac troponin T; *MYL2*: myosin light chain 2; *MYL3*: myosin light chain 3; *ALPK3*: alpha-protein kinase 3; TTNtv: titin truncating variants; DCM: dilated cardiomyopathy; *LMNA*: lamin A and lamin C proteins; ACM: arrhythmogenic cardiomyopathy; *RBM20*: RNA binding motif protein 20; FLNCtv: filamin C truncating variants; *PKP2*: plakophilin-2; *PLN*: phospholamban; *DSG2*: desmoglein-2.

**Gene**	**Cardiomyopathy**	**Estimated Penetrance**	**Reference**
*MYBPC3*	HCM	50–60%	Topriceanu, C.C., *Circulation*, 2024 [13]
*MYH7*	HCM	50–75%	Topriceanu, C.C., *Circulation*, 2024 [13]
*TNNT2*	HCM	40–80%	Topriceanu, C.C., *Circulation*, 2024 [13]
*MYL2*	HCM	30–90%	Topriceanu, C.C., *Circulation*, 2024 [13]
*MYL3*	HCM	10–70%	Topriceanu, C.C., *Circulation*, 2024 [13]
*ALPK3*	HCM	30–70%	Topriceanu, C.C., *Circulation*, 2024 [13]
TTNtv	DCM	13–17% (incidental finding)	Cabrera-Romero, E., *JACC*, 2024 [14]
TTNtv	DCM	40–60% (familial context)	Mazzarotto, F., *Circulation*, 2020 [3]
*LMNA*	DCM/ACM	70–100%	Hasselberg, N.E., *Eur Heart J*, 2018 [15]
*RBM20*	DCM	60–90%	Parikh, V.N., *Circ Heart Fail*, 2019 [16]
FLNCtv	DCM	97%	Ortiz-Genga, M.F., *JACC*, 2016 [17]
*PKP2*	ACM	50–80%	Dalal, D., *JACC*, 2006 [18]
*PLN* (R14del)	DCM / ACM	50–90%	Verstraelen, T.E., *Eur J Heart Fail*, 2025 [19]
*DSG2*	ACM	58–75%	Syrris, P., *Eur Heart J*, 2007 [20]

**Table 2:** Sarcomeric variants with evidence of intermediate effects. *MYBPC3*: myosin-binding protein C; *MYH7*: beta-myosin heavy chain; *TNNT2*: Cardiac troponin T; *MYL3*: myosin light chain 3; *TNNI3*: cardiac troponin I.

Gene	Genomic Variant	Protein Variant	Reference
*MYBPC3*	c.2429G>A	p.Arg810His	Meisner, J. K., *Circulation*, 2025 [10]
*MYH7*	c.3981C>A	p.Asn1327Lys	Meisner, J. K., *Circulation*, 2025 [10]
*MYBPC3*	c.442G>A	p.Gly148Arg	Meisner, J. K., *Circulation*, 2025 [10]
*MYBPC3*	c.1224-52G>A	----	Meisner, J. K., *Circulation*, 2025 [10]
*MYH7*	c.976G>C	p.Ala326Pro	Meisner, J. K., *Circulation*, 2025 [10]
*TNNT2*	c.832C>T	p.Arg278Cys	Meisner, J. K., *Circulation*, 2025 [10]
*MYBPC3*	c.1828G>C	p.Asp610His	Meisner, J. K., *Circulation*, 2025 [10]
*MYL3*	c.170C>A	p.Ala57Asp	Meisner, J. K., *Circulation*, 2025 [10]
*MYBPC3*	c.3065G>C	p.Arg1022Pro	Meisner, J. K., *Circulation*, 2025 [10]
*MYBPC3*	c.2618C>A	p.Pro873His	Meisner, J. K., *Circulation*, 2025 [10]
*MYBPC3*	c.1321G>A	p.Glu441Lys	Meisner, J. K., *Circulation*, 2025 [10]
*MYBPC3*	c.1813G>A	p.Asp605Asn	Meisner, J. K., *Circulation*, 2025 [10]
*TNNT2*	c.887G>A	p.R286H	Pua, C. J., *Circ, Genom. Precis. Med*, 2020 [45]
*TNNI3*	c.235C>T	p.R79C	Pua, C. J., *Circ, Genom. Precis. Med*, 2020 [45]
*TNNT2*	c.842A>T	p.Asn271Ile	Larrañaga-Moreira, J.M., *JACC HF*, 2025 [46]

## Data Availability

No new data were analyzed in this study. Data sharing is not applicable to this article.

## Supplementary Materials

The following supporting information can be downloaded at: https://www.mdpi.com/article/10.3390/jcdd12070274/s1, Table S1. Common variants associated with hypertrophic cardiomyopathy (HCM) identified through genome-wide association studies (GWAS). Table S2. Common variants associated with cardiac structure and function traits identified through genome-wide association studies (GWAS).
