# Supplementary material for: From Rare Genetic Variants to Polygenic Risk: Understanding the Genetic Basis of Cardiomyopathies
Source: J Cardiovasc Dev Dis. 2025 Jul 17;12(7):274. doi: 10.3390/jcdd12070274 (PMC12294854; doi:10.3390/jcdd12070274)
Supplement: Supplementary file 1 [file jcdd-12-00274-s001.zip › jcdd-3702666-supplementary.pdf]

**Table S1. Common variants associated with hypertrophic cardiomyopathy (HCM) identified through genome-wide association studies (GWAS)**. BAG3: BCL2-associated athanogene 3; FHOD3: Formin homology 2 domain containing 3; TPGS2: Tubulin polyglutamylase complex subunit 2; MYBPC3: Myosin-binding protein C, cardiac-type; HSPB7: Heat shock protein beta-7; VPREB3: Pre-B lymphocyte 3; SMARCB1: SWI/SNF-related, matrix-associated, actin-dependent regulator of chromatin subfamily B member 1; CDKN1A: Cyclin-dependent kinase inhibitor 1A (p21, Cip1); SLC35F1: Solute carrier family 35 member F1; PLN: Phospholamban; PRKCA: Protein kinase C alpha; CRHR1: Corticotropin-releasing hormone receptor 1; MAPT: Microtubule-associated protein tau; ALPK3: Alpha kinase 3; NMB: Neuromedin B; VTI1A: Vesicle transport through interaction with t-SNAREs 1A; TCF7L2: Transcription factor 7-like 2; TBX3: T-box transcription factor 3; MYOZ1: Myozenin 1; SYNPO2L: Synaptopodin 2-like; ADPRHL1: ADP-ribosylhydrolase like 1; ACTBL2: Actin beta-like 2; FNDC3B: Fibronectin type III domain containing 3B; SLC6A6: Solute carrier family 6 member 6 (taurine transporter); LSM3: LSM3 homolog, U6 small nuclear RNA associated; STRN: Striatin; GSC: Goosecoid homeobox; SPATA24: Spermatogenesis associated 24; CCDC136: Coiled-coil domain containing 136; FLNC: Filamin C; DMPK: Dystrophia myotonica protein kinase; SYMPK: Symplekin; MTSS1: Metastasis suppressor 1; CCDC141: Coiled-coil domain containing 141; SESTD1: SEC14 and spectrin domain containing 1; XPO7: Exportin 7; RRAS2: RAS-related protein R-Ras2; COPB1: Coatomer protein complex subunit beta 1; CCT8: Chaperonin containing TCP1 subunit 8; E2F6: E2F transcription factor 6; ROCK2: Rho-associated coiled-coil containing protein kinase 2; TRDN: Triadin; HEY2: Hes-related family bHLH transcription factor with YRPW motif 2; TMEM182: Transmembrane protein 182; MFSD9: Major facilitator superfamily domain containing 9; NEDD4L: Neural precursor cell expressed, developmentally down-regulated 4-like; CHPF: Chondroitin polymerizing factor; KLF6: Kruppel-like factor 6; AKR1E2: Aldo-keto reductase family 1 member E2; FERMT2: Fermitin family member 2 (Kindlin-2); ERO1A: Endoplasmic reticulum oxidoreductase 1 alpha.

| Locus / SNP | Nearest Gene(s) | Estimated Effect | Notes | Reference |
|---|---|---|---|---|
| rs72840788 | *BAG3* | OR 1.52 | Sarcomere co-chaperone | Harper et al., *Nat Genet*, 2021[8] |
| rs28768976 | *SPPL2C* | OR 1.29 | Intramembrane protease | Harper et al., *Nat Genet,* 2021[8] |
| rs7210446 | *PRKCA* | OR 1.25 | Cardiac kinase | Harper et al., *Nat Genet*, 2021[8] |
| rs4799426 | *FHOD3* | OR 1.38 | Sarcomere formin | Harper et al., *Nat Genet*, 2021[8] |
| rs118060942 | *FHOD3* | OR 1.79 | Sarcomere formin | Harper et al., *Nat Genet*, 2021[8] |
| rs2234962 | *BAG3* | OR 1.45 | Sarcomere co-chaperone | Tadros et al., *Nat Genet*, 2025[9] |
| rs2644262 | *FHOD3/TPGS2* | OR 1.38 | Sarcomere formin | Tadros et al., *Nat Genet*, 2025[9] |
| rs78310129 | *MYBPC3* | OR 3.53 | Sarcomeric protein | Tadros et al., *Nat Genet*, 2025[9] |
| rs1048302 | *HSPB7* | OR 1.28 | Heat shock protein | Tadros et al., *Nat Genet*, 2025[9] & Harper et al., *Nat Genet,* 2021[8] |
| rs2070458 | *VPREB3/SMARCB1* | OR 1.30 | Chromatin remodeler | Tadros et al., *Nat Genet*, 2025[9] & Harper et al., *Nat Genet,* 2021[8] |
| rs3176326 | *CDKN1A* | OR 1.30 | Cell cycle regulator | Tadros et al., *Nat Genet*, 2025[9] & Harper et al., *Nat Genet,* 2021[8] |
| rs12212795 | *SLC35F1/PLN* | OR 1.51 | Calcium handling protein | Tadros et al., *Nat Genet*, 2025[9] & Harper et al., *Nat Genet,* 2021[8] |
| rs4577128 | *PRKCA* | OR 1.23 | Protein kinase C alpha | Tadros et al., *Nat Genet*, 2025[9] |
| rs393838 | *CRHR1/MAPT* | OR 1.26 | CRHR1: stress protein; MAPT: microtubules | Tadros et al., *Nat Genet*, 2025[9] |
| rs8033459 | *ALPK3/NMB* | OR 1.20 | Sarcomere kinase | Tadros et al., *Nat Genet*, 2025[9] & Harper et al., *Nat Genet,* 2021[8] |
| rs11196085 | *VTI1A/TCF7L2* | OR 1.22 | Transcription factor | Tadros et al., *Nat Genet*, 2025[9] |
| rs7301677 | *TBX3* | OR 1.22 | Transcription factor | Tadros et al., *Nat Genet*, 2025[9] & Harper et al., *Nat Genet,* 2021[8] |
| rs2177843 | *MYOZ1/SYNPO2L* | OR 1.26 | Z-disc scaffold protein | Tadros et al., *Nat Genet*, 2025[9] |
| rs41306688 | *ADPRHL1* | OR 1.60 | Actin-binding protein | Tadros et al., *Nat Genet*, 2025[9] & Harper et al., *Nat Genet,* 2021[8] |
| rs2191445 | *ACTBL2* | OR 1.23 | Beta-actin-like protein | Tadros et al., *Nat Genet*, 2025[9] |
| rs4894803 | *FNDC3B* | OR 1.18 | Fibronectin domain protein | Tadros et al., *Nat Genet*, 2025[9] |
| rs13061705 | *SLC6A6/LSM3* | OR 1.19 | Taurine transporter | Tadros et al., *Nat Genet*, 2025[9] & Harper et al., *Nat Genet,* 2021[8] |
| rs13021775 | *STRN* | OR 1.17 | Calmodulin-binding protein | Tadros et al., *Nat Genet*, 2025[9] |
| rs8006225 | *GSC* | OR 1.22 | Goosecoid homeobox protein | Tadros et al., *Nat Genet*, 2025[9] |
| rs10052399 | *SPATA24* | OR 1.18 | Unknown function | Tadros et al., *Nat Genet*, 2025[9] |
| rs66520020 | *CCDC136/FLNC* | OR 1.21 | Sarcomeric protein | Tadros et al., *Nat Genet*, 2025[9] |

| | | | | |
|---|---|---|---|---|
| rs12460541 | *DMPK/SYMPK* | OR 1.16 | Protein kinase | Tadros et al., *Nat Genet*, 2025[9] |
| rs7461129 | *MTSS1* | OR 1.16 | Actin-binding protein | Tadros et al., *Nat Genet*, 2025[9] |
| rs56005624 | *CCDC141/SESTD1* | OR 1.21 | Coiled-coil domain protein | Tadros et al., *Nat Genet*, 2025[9] |
| rs7824244 | *XPO7* | OR 1.22 | Nuclear export factor | Tadros et al., *Nat Genet*, 2025[9] |
| rs12270374 | *RRAS2/COPB1* | OR 1.14 | Ras-related protein | Tadros et al., *Nat Genet*, 2025[9] |
| rs62222424 | *CCT8* | OR 1.32 | Chaperonin complex protein | Tadros et al., *Nat Genet*, 2025[9] |
| rs11687178 | *E2F6/ROCK2* | OR 1.14 | E2F6: cell cycle repressor; ROCK2: cytoskeleton regulator | Tadros et al., *Nat Genet*, 2025[9] |
| rs9320939 | *TRDN/HEY2* | OR 1.13 | Transcriptional repressor | Tadros et al., *Nat Genet*, 2025[9] |
| rs2540277 | *TMEM182/MFSD9* | OR 1.32 | Muscle differentiation protein | Tadros et al., *Nat Genet*, 2025[9] |
| rs6566955 | *NEDD4L* | OR 1.14 | Ubiquitin ligase | Tadros et al., *Nat Genet*, 2025[9] |
| rs13004994 | *CHPF* | OR 1.13 | Chondroitin polymerizing factor | Tadros et al., *Nat Genet*, 2025[9] |
| rs2645210 | *KLF6/AKR1E2* | OR 1.16 | Transcription factor | Tadros et al., *Nat Genet*, 2025[9] |
| rs113907726 | *FERMT2/ERO1A* | OR 1.16 | Integrin signaling protein | Tadros et al., *Nat Genet*, 2025[9] |

**Table S2. Common variants associated with cardiac structure and function traits identified through ge-nome-wide association studies (GWAS)**. LVEDV: left ventricular end-diastolic volume; LVEDVi: LV end-diastolic volume indexed; LVEF: LV ejection fraction; LVESV: LV end-systolic volume; LVESVi: LV end-systolic volume indexed; SV: stroke volume; SVi: stroke volume indexed. SNPs, nearest genes, inverse-normalized beta coefficient (InvNorm Beta), and functional annotations are provided as reported by Pirruccello et al. [34]. Gene functions are summarized from biological databases and literature (https://www.omim.org/), with emphasis on cardiac relevance.

| Trait | dbSNP | Nearest Gene | InvNorm Beta | Notes |
|---|---|---|---|---|
| **LVEDV** | | | | |
| | rs28579893 | *CLCNKA* | -0,037 | Renal chloride channel; indirectly linked to blood pressure and cardiac load regulation |
| | rs753562515 | *AKR1A1* | -0,035 | Aldo-keto reductase; involved in detoxification and oxidative stress, potential indirect cardiac role |
| | rs7605066 | *ZNF638* | 0,033 | Zinc finger transcription factor; implicated in adipogenesis and metabolic regulation |
| | rs539762056 | *SP3* | 0,035 | Transcription factor involved in gene regulation during development and possibly cardiac remodeling |
| | rs1873164 | *CCDC141* | -0,06 | Coiled-coil domain protein associated with heart rate and sinoatrial node function |
| | rs73028849 | *XPC* | 0,04 | DNA repair protein involved in nucleotide excision repair; no direct cardiac role established |
| | rs6777123 | *MECOM* | 0,034 | Transcriptional repressor; unclear role in heart |
| | rs4521636 | *HLA-B* | 0,039 | MHC class I gene; involved in immune response and possibly cardiac inflammation |
| | rs9275587 | *HLA-DQA2* | 0,037 | MHC class II gene; involved in antigen presentation and potential immune-mediated cardiac injury |
| | rs2146324 | *VEGFA* | 0,039 | Cell cycle inhibitor; regulates cardiomyocyte proliferation |
| | rs11153730 | *PLN* | -0,048 | Regulator of sarcoplasmic calcium uptake; associated with dilated and arrhythmogenic cardiomyopathies |
| | rs3918226 | *NOS3* | 0,058 | Endothelial nitric oxide synthase; modulates vascular tone and myocardial function |
| | rs72840788 | *BAG3* | 0,055 | Sarcomeric co-chaperone; involved in protein quality control and cardiomyocyte survival |
| | rs7306710 | *LLPH* | 0,039 | Nuclear protein with poorly characterized function; limited evidence in cardiac tissue |
| | rs3184504 | *SH2B3* | -0,048 | Cytokine signaling adaptor (LNK); associated with cardiovascular traits and inflammation |
| | rs10850034 | *HECTD4* | 0,05 | E3 ubiquitin ligase; associated with metabolic and cardiovascular traits |
| | rs71385734 | *PKD1* | 0,05 | Mechanosensory transmembrane protein; associated with vascular and structural heart abnormalities |
| | rs2302455 | *MYO1C* | 0,059 | Unconventional myosin; involved in intracellular trafficking, limited cardiac evidence |
| | rs12460541 | *RSPH6A* | -0,04 | Ciliary protein; no known role in cardiac tissue |
| **LVEDi** | | | | |
| | rs1976402 | *SPEN* | 0,043 | Transcriptional repressor involved in Notch signalling; limited cardiac evidence |

| | | | | |
|---|---|---|---|---|
| | rs1873164 | *CCDC141* | -0,07 | Coiled-coil domain protein associated with heart rate and sinoatrial node function |
| | rs767987273 | *SPATS2L* | 0,04 | RNA-binding protein; associated with blood pressure and cardiac traits in GWAS |
| | rs73028849 | *XPC* | 0,044 | DNA repair protein involved in nucleotide excision repair; no direct cardiac role established |
| | rs12499670 | *HAND2* | 0,04 | Cardiac transcription factor essential for right ventricular and outflow tract development |
| | rs6458349 | *VEGFA* | 0,044 | Cell cycle inhibitor; regulates cardiomyocyte proliferation |
| | rs9480737 | *BEND3* | -0,043 | Transcriptional repressor; limited data on cardiac or metabolic roles |
| | rs72967533 | *PLN* | -0,049 | Regulator of sarcoplasmic calcium uptake; associated with dilated and arrhythmogenic cardiomyopathies |
| | rs72840788 | *BAG3* | 0,059 | Sarcomeric co-chaperone; involved in protein quality control and cardiomyocyte survival |
| | rs35350651 | *ATXN2* | -0,044 | RNA-binding protein; associated with cellular stress responses and neurodegeneration |
| | rs10850034 | *HECTD4* | 0,042 | E3 ubiquitin ligase; associated with metabolic and cardiovascular traits |
| | rs7502466 | *MYO1C* | 0,063 | Unconventional myosin; involved in intracellular trafficking, limited cardiac evidence |
| | rs9797817 | *RSPH6A* | -0,051 | Ciliary protein; no known role in cardiac tissue |
| **LVEF** | | | | |
| | rs2503715 | *C1orf86* | -0,058 | Uncharacterized protein; no known role in cardiac tissue |
| | rs1739837 | *HSPB7* | 0,071 | Small heat shock protein; protects against sarcomeric damage in cardiac stress |
| | rs10925197 | *ACTN2* | 0,042 | Sarcomeric actin-binding protein; linked to inherited cardiomyopathies |
| | rs2562845 | *TTN* | -0,079 | Giant sarcomeric protein; major genetic cause of dilated cardiomyopathy |
| | rs11710541 | *XPC* | -0,065 | DNA repair protein involved in nucleotide excision repair; no direct cardiac role established |
| | rs56099248 | *MITF* | -0,061 | Transcription factor; limited evidence for role in cardiac development |
| | rs35999985 | *DNAJC18* | 0,048 | Hsp40 family protein; unclear cardiac relevance |
| | rs9274626 | *HLA-DQB1* | -0,042 | MHC class II gene; immune response and potential link to myocarditis |
| | rs3176326 | *CDKN1A* | -0,078 | Cell cycle inhibitor (p21); regulates cardiomyocyte proliferation and stress response |
| | rs3807309 | *FLNC* | -0,083 | Sarcomeric protein; mutations cause various forms of inherited cardiomyopathy |
| | rs36029352 | *DEFB136* | -0,038 | Antimicrobial defensin; no known role in cardiac function |
| | rs4073554 | *PTK2* | 0,043 | Focal adhesion kinase; regulates cardiac response to mechanical stress and remodelling |
| | rs189569984 | *RBM20* | -0,201 | RNA splicing regulator; key modulator of titin isoforms and cause of dilated cardiomyopathy |

| | | | | |
|---|---|---|---|---|
| | rs72840788 | *BAG3* | -0,1 | Sarcomeric co-chaperone; involved in protein quality control and cardiomyocyte survival |
| | rs721067 | *CSRP3* | 0,07 | LIM domain sarcomeric protein; associated with hypertrophic cardiomyopathy |
| | rs113819537 | *SSPN* | 0,045 | Sarcospan; membrane protein involved in muscular dystrophies and cardiac membrane stability |
| | rs8023658 | *ZNF592* | -0,046 | Zinc finger protein; putative transcriptional regulator, limited cardiac data |
| | rs5029142 | *LMF1* | -0,043 | Lipase maturation factor; associated with lipid metabolism and cardiovascular risk |
| | rs12452367 | *HLF* | -0,055 | Transcription factor; regulates circadian and metabolic pathways, potential cardiac effects |
| | rs2047273 | *FHOD3* | -0,042 | Cardiac formin involved in sarcomere organization; associated with hypertrophic cardiomyopathy |
| | rs10871753 | *NEDD4L* | -0,043 | E3 ubiquitin ligase; regulates ion channels and cardiac electrical activity |
| | rs2070458 | *SMARCB1* | 0,063 | Chromatin remodelling factor; unclear cardiac role |
| **LVESV** | | | | |
| | rs114300540 | *RPL22* | -0,059 | Ribosomal protein; no established role in cardiac disease |
| | rs1048302 | *HSPB7* | -0,06 | Small heat shock protein; protects against sarcomeric damage in cardiac stress |
| | rs753562515 | *AKR1A1* | -0,037 | Aldo-keto reductase; involved in detoxification and oxidative stress, potential indirect cardiac role |
| | rs2562845 | *TTN* | 0,077 | Giant sarcomeric protein; major genetic cause of dilated cardiomyopathy |
| | rs11710541 | *XPC* | 0,058 | DNA repair protein involved in nucleotide excision repair; no direct cardiac role established |
| | rs13092177 | *EPHB1* | 0,048 | Receptor tyrosine kinase; possibly involved in cardiac signalling |
| | rs1499813 | *FNDC3B* | 0,035 | Fibronectin domain protein; potential role in cell adhesion |
| | rs9274626 | *HLA-DQB1* | 0,039 | MHC class II gene; immune response and potential link to myocarditis |
| | rs730506 | *CDKN1A* | 0,053 | Cell cycle inhibitor (p21); regulates cardiomyocyte proliferation and stress response |
| | rs11153730 | *PLN* | -0,038 | Regulator of sarcoplasmic calcium uptake; associated with cardiomyopathy |
| | rs34373805 | *FLNC* | 0,055 | Sarcomeric protein; mutations cause various forms of inherited cardiomyopathy |
| | rs1962104 | *AGO2* | -0,037 | Argonaute protein; miRNA regulation, cardiac remodelling link |
| | rs72840788 | *BAG3* | 0,087 | Sarcomeric co-chaperone; involved in protein quality control and cardiomyocyte survival |
| | rs10832164 | *RRAS2* | -0,033 | RAS family GTPase; involved in cardiac signalling |
| | rs11604807 | *CSRP3* | -0,049 | LIM domain sarcomeric protein; associated with hypertrophic cardiomyopathy |
| | rs113819537 | *SSPN* | -0,04 | Sarcospan; membrane protein involved in muscular dystrophies and cardiac membrane stability |

| | | | | |
|---|---|---|---|---|
| | rs3184504 | *SH2B3* | -0,039 | Cytokine signalling adaptor (LNK); associated with cardiovascular traits and inflammation |
| | rs10850034 | *HECTD4* | 0,041 | E3 ubiquitin ligase; associated with metabolic and cardiovascular traits |
| | rs116904997 | *PXN* | -0,112 | Paxillin; focal adhesion protein, possible role in cardiac stress response |
| | rs3829491 | *LMF1* | 0,034 | Lipase maturation factor; associated with lipid metabolism and cardiovascular risk |
| | rs71385734 | *PKD1* | 0,053 | Mechanosensory transmembrane protein; associated with vascular and structural heart abnormalities |
| | rs2302455 | *MYO1C* | 0,063 | Unconventional myosin; involved in intracellular trafficking, limited cardiac evidence |
| | rs242562 | *MAPT* | -0,037 | Tau protein; mainly neuronal, cardiac role under investigation |
| | rs12452367 | *HLF* | 0,047 | Transcription factor; regulates circadian and metabolic pathways, potential cardiac effects |
| | rs9897002 | *PRKCA* | 0,033 | Protein kinase C alpha; involved in cardiac signalling and hypertrophy |
| | rs10871753 | *NEDD4L* | 0,033 | E3 ubiquitin ligase; regulates ion channels and cardiac electrical activity |
| | rs16975238 | *ATP5SL* | -0,034 | ATP synthase-related protein; mitochondrial function |
| | rs10421891 | *RSPH6A* | -0,045 | Ciliary protein; no known role in cardiac tissue |
| | rs5760061 | *DERL3* | -0,055 | ER-associated degradation protein; no established cardiac role |
| **LVESi** | | | | |
| | rs709208 | *RNF207* | -0,041 | Ring finger protein; associated with cardiac conduction traits in GWAS |
| | rs945425 | *CLCNKA* | -0,07 | Renal chloride channel; indirectly linked to blood pressure and cardiac load regulation |
| | rs753562515 | *AKR1A1* | -0,038 | Aldo-keto reductase; involved in detoxification and oxidative stress, potential indirect cardiac role |
| | rs2562845 | *TTN* | 0,085 | Giant sarcomeric protein; major genetic cause of dilated cardiomyopathy |
| | rs190093681 | *SESTD1* | 0,374 | SEC14 domain protein; implicated in calcium signalling and atrial fibrillation |
| | rs774290282 | *SPATS2L* | -0,038 | RNA-binding protein; associated with blood pressure and cardiac traits in GWAS |
| | rs73028849 | *XPC* | 0,064 | DNA repair protein involved in nucleotide excision repair; no direct cardiac role established |
| | rs79502300 | *MITF* | 0,054 | Transcription factor; limited evidence for role in cardiac development |
| | rs2886037 | *MLF1* | -0,036 | Myeloid leukemia factor 1; nuclear protein, cardiac role unclear |
| | rs1499813 | *FNDC3B* | 0,04 | Fibronectin domain protein; potential role in cell adhesion |
| | rs11748963 | *PROB1* | 0,044 | Proline-rich protein; function not well characterized in cardiac tissue |
| | rs3176326 | *CDKN1A* | 0,065 | Cell cycle inhibitor (p21); regulates cardiomyocyte proliferation and stress response |
| | rs34373805 | *FLNC* | 0,067 | Sarcomeric protein; mutations cause various forms of |

| | | | | |
|---|---|---|---|---|
| | | | | inherited cardiomyopathy |
| | rs1962104 | *AGO2* | -0,043 | Argonaute protein; miRNA regulation, cardiac remodelling link |
| | rs189569984 | *RBM20* | 0,214 | RNA splicing regulator; key modulator of titin isoforms and cause of dilated cardiomyopathy |
| | rs72840788 | *BAG3* | 0,093 | Sarcomeric co-chaperone; involved in protein quality control and cardiomyocyte survival |
| | rs11023059 | *RRAS2* | 0,042 | RAS family GTPase; involved in cardiac signalling |
| | rs116904997 | *PXN* | -0,132 | Paxillin; focal adhesion protein, possible role in cardiac stress response |
| | rs8063213 | *LMF1* | 0,045 | Lipase maturation factor; associated with lipid metabolism and cardiovascular risk |
| | rs2302455 | *MYO1C* | 0,065 | Unconventional myosin; involved in intracellular trafficking, limited cardiac evidence |
| | rs242562 | *MAPT* | -0,04 | Tau protein; mainly neuronal, potential role in cardiac aging and stress response |
| | rs12452367 | *HLF* | 0,055 | Transcription factor; regulates circadian and metabolic pathways, potential cardiac effects |
| | rs9892651 | *PRKCA* | 0,04 | Protein kinase C alpha; involved in cardiac signalling and hypertrophy |
| | rs10871753 | *NEDD4L* | 0,039 | E3 ubiquitin ligase; regulates ion channels and cardiac electrical activity |
| | rs10421891 | *RSPH6A* | -0,053 | Ciliary protein; no known role in cardiac tissue |
| | rs5760061 | *DERL3* | -0,064 | ER-associated degradation protein; limited evidence for cardiac involvement |
| **SV** | | | | |
| | rs7573293 | *CCDC141* | -0,049 | Coiled-coil domain protein associated with heart rate and sinoatrial node function |
| | rs888690 | *NKX2-5* | -0,039 | Cardiac transcription factor; essential for heart development and conduction |
| | rs111721712 | *HLA-B* | 0,038 | MHC class I gene; immune response, possibly linked to myocarditis or rejection |
| | rs28391274 | *HLA-DQB1* | 0,047 | MHC class II gene; immune response and potential link to myocarditis |
| | rs2146324 | *VEGFA* | 0,041 | Cell cycle inhibitor; regulates cardiomyocyte proliferation |
| | rs72967533 | *PLN* | -0,051 | Regulator of sarcoplasmic calcium uptake; associated with cardiomyopathy |
| | rs10400419 | *LLPH* | 0,04 | Nuclear protein with poorly characterized function; limited evidence in cardiac tissue |
| | rs11065979 | *ATXN2* | 0,048 | RNA-binding protein; associated with cellular stress responses and neurodegeneration |
| | rs11066188 | *HECTD4* | 0,045 | E3 ubiquitin ligase; associated with metabolic and cardiovascular traits |
| | rs2891403 | *RPH3A* | -0,042 | Synaptic vesicle protein; no known role in cardiomyocytes |
| | rs422068 | *MYH6* | 0,039 | Alpha-myosin heavy chain; essential for atrial contraction and cardiac conduction |
| | rs143384 | *GDF5* | -0,035 | Growth differentiation factor; involved in development, possible cardiac effects |

| | | | |
|---|---|---|---|
| **SVi** | | | |
| rs7573293 | *CCDC141* | -0,051 | Coiled-coil domain protein associated with heart rate and sinoatrial node function |
| rs6458349 | *VEGFA* | 0,045 | Cell cycle inhibitor; regulates cardiomyocyte proliferation |
| rs9480737 | *BEND3* | -0,047 | Transcriptional repressor; limited data on cardiac or metabolic roles |
| rs72967533 | *PLN* | -0,048 | Regulator of sarcoplasmic calcium uptake; associated with cardiomyopathy |
| rs1919865 | *GJA1* | 0,06 | Connexin 43; key gap junction protein involved in cardiac conduction |
| rs579459 | *SURF6* | -0,05 | Ribosome biogenesis factor; cardiac role not established |
| rs376439 | *MYH6* | 0,043 | Alpha-myosin heavy chain; essential for atrial contraction and cardiac conduction |
